# Accurate recording of personality disorder in clinical practice

**DOI:** 10.1192/bjb.2018.40

**Published:** 2018-08

**Authors:** Peter Tyrer

**Affiliations:** Centre for Psychiatry, Imperial College, London, UK

## Abstract

Disturbances of personality are recorded very poorly in official statistics, but there are signs that this is changing. For many years, personality disorder has been either regarded as a secondary diagnosis that can be forgotten in the presence of another mental disorder, or avoided as the diagnosis gives the impression of untreatability or stigma. What is now abundantly clear is that under-diagnosis of personality disorder represents a disservice to patients and practitioners. It prevents a proper understanding of the longitudinal course of psychiatric disorder and an appreciation of some of the positive aspects of abnormal personality that can be used in treatment. We must no longer bury personality disorder, ostrich-like, in the diagnostic sand. It is there for the asking and needs to be embraced honestly and without fear if we are to improve the management of psychiatric patients.

**Declaration of interest:**

None.

The paper by Hossain *et al*^1^ in this issue is noteworthy for two reasons: it records ethnic variation in a sensitive subject and provides a rare longitudinal record of personality disorder diagnosis. This type of research, based in clinical practice, should help to destigmatise the diagnosis of personality disorder, which for many years has been under-recorded in formal statistics. There is something bizarre in the contradiction between the research data, showing prevalence figures of up to 40% in psychiatric in-patients^2^^,^^3^ (and much higher for those in tertiary services)^4^ and the official national diagnostic figures, which rarely exceed 8%.^5^ This difference can only be explained by either (a) failure of detection; (b) diagnostic avoidance for a number of reasons; or (c) observance of a separate axis for personality disorder, one of the advantages of the DSM system that has now been lost.^6^ I would like to think that the fourth option, a complete rejection of the diagnosis of personality disorder, is not currently embraced.

It is likely that all three may be relevant in under-diagnosis and readers might ask themselves how they normally avoid this diagnosis in practice. One of the reasons may be the perceived lack of utility of the diagnosis. Does it help clinical practice? Many feel it does not as it is felt to confer an unfair label of untreatability; but this is mistaken. Three-quarters of those with personality disorder admitted to UK psychiatric hospitals are given the diagnosis of emotionally unstable (borderline) personality disorder,^5^ and this has the best evidence base for treatment.^7^ Lack of treatment options may be a reason for the low diagnosis rates of other personality disorders – anankastic personality disorder only accounts for 0.18% of all diagnoses in the group^5^ – but this does not mean diagnoses of personality disorders other than borderline are of no therapeutic value.^8^

Personality dysfunction may also be an advantage in aiding the effectiveness of certain forms of treatment^9^^,^^10^ and such findings, if confirmed in other settings, would help greatly in destigmatising the disorder. Hossain *et al*^1^ also report a high rate of diagnosis in adolescence. The new ICD-11 diagnostic classification of personality disorder, to be introduced later in 2018, includes the diagnostic option of ‘personality disorder in development’,^11^ and this will allow clinicians to make this diagnosis in younger people. This does not mean that a diagnosis made at this time becomes an indelible stain on a person's mental health; it merely states that, at that particular time, the individual concerned has significant personality dysfunction and this should be acknowledged instead of reducing every form of pathology to symptoms or behaviour.

The low rate of diagnosis in Black and minority ethnic populations^1^ can probably be explained by what could be called ‘compensatory stigma’. Of the three prevalence studies of personality disorder in ethnic minorities, two have shown reduced prevalence compared with White comparators^1^^,^^12^ but the other, assessed as part of a national survey, showed an increase.^13^ My view is that the national survey is nearer to the truth. There is a concern that a psychiatric diagnosis of personality disorder in certain ethnic minority groups might be construed as racist and so is avoided. I have certainly behaved like this in my own diagnostic practice in the past.

The increased prevalence of personality disorder over time shown by Hossain *et al*^1^ should not be regarded as necessarily a true reflection of increase; rather it shows that clinicians may be less wary about making the diagnosis that they previously did. This may well be good for practice, as assessment of personality as well as mental health status makes for better understanding and broader predictive value. The ICD-11 classification may increase prevalence rates of personality disorder^3^ as it allows for the diagnosis to be made for the first time both earlier and later in life.^11^

Further studies along the same lines as Hossain *et al*^1^ should also examine the proportion of people admitted with personality disorder under the Mental Health Act. Those with personality disorder are sectioned less often after formal assessment than those with other diagnoses, but at 41% the proportion is still substantial^14^ and may be increasing. This certainly appears to be the case in those with personality dysfunction and intellectual disability^15^ and is a matter of some concern, as in this population the diagnosis of personality disorder is more contentious. It should also help to have a simpler diagnostic system that clarifies the difference between severe and milder forms of personality disorder; the severe level is actually rare.

## About the author

**Peter Tyrer** is Emeritus Professor of Psychiatry at the Centre for Psychiatry, Imperial College, London. He was the Chair of the World Health Organization ICD-11 Revision Group for Personality Disorders between 2010 and 2017.
